# *tert*-Butyl 2-borono-1*H*-pyrrole-1-carboxyl­ate

**DOI:** 10.1107/S1600536809007375

**Published:** 2009-03-06

**Authors:** Zheng Zhong, Guo-Qiang Lin, Zhi-Hua Sun, Bing Wang

**Affiliations:** a Department of Chemistry, Fudan University, 220 Handan Road, Shanghai, 200433, People’s Republic of China

## Abstract

In the crystal structure of the title compound, C_9_H_14_BNO_4_, the boronic acid group and carbamate groups are nearly co-planar with the pyrrole ring, making dihedral angles of 0.1 (2) and 2.2 (2)°, respectively. Intra­molecular and inter­molecular O—H⋯O hydrogen bonds help to stabilize the structure, the latter interaction leading to inversion dimers..

## Related literature

For general background, see: Hall (2005 ▶); Kelly & Fuchs (1993 ▶).
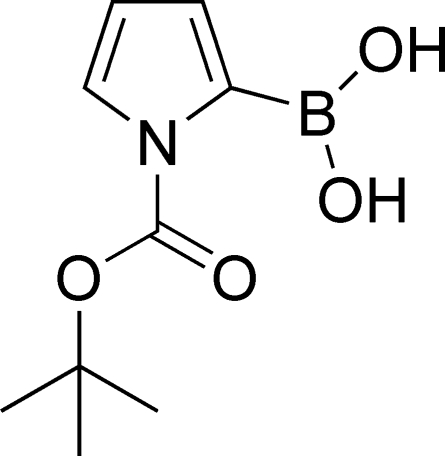


## Experimental

### 

#### Crystal data

C_9_H_14_BNO_4_
*M* *_r_* = 211.02
Orthorhombic, 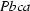

*a* = 13.014 (3) Å
*b* = 9.940 (2) Å
*c* = 17.417 (4) Å
*V* = 2252.9 (9) Å^3^
*Z* = 8
Mo *K*α radiation
μ = 0.10 mm^−1^
*T* = 293 K
0.25 × 0.12 × 0.10 mm

#### Data collection

Bruker SMART 1000 CCD area-detector diffractometer
Absorption correction: none
9542 measured reflections
2213 independent reflections
1208 reflections with *I* > 2σ(*I*)
*R*_int_ = 0.065

#### Refinement

*R*[*F* ^2^ > 2σ(*F* ^2^)] = 0.042
*wR*(*F* ^2^) = 0.106
*S* = 0.85
2213 reflections
192 parameters
All H-atom parameters refined
Δρ_max_ = 0.12 e Å^−3^
Δρ_min_ = −0.21 e Å^−3^

### 

Data collection: *SMART* (Bruker, 2001 ▶); cell refinement: *SAINT* (Bruker, 2001 ▶); data reduction: *SAINT*; program(s) used to solve structure: *SHELXTL* (Sheldrick, 2008 ▶); program(s) used to refine structure: *SHELXTL*; molecular graphics: *SHELXTL*; software used to prepare material for publication: *SHELXTL*.

## Supplementary Material

Crystal structure: contains datablocks I, New_Global_Publ_Block. DOI: 10.1107/S1600536809007375/xu2484sup1.cif

Structure factors: contains datablocks I. DOI: 10.1107/S1600536809007375/xu2484Isup2.hkl

Additional supplementary materials:  crystallographic information; 3D view; checkCIF report

## Figures and Tables

**Table 1:** Hydrogen-bond geometry (Å, °)

*D*—H⋯*A*	*D*—H	H⋯*A*	*D*⋯*A*	*D*—H⋯*A*
O3—H3*A*⋯O2	0.87 (2)	1.73 (2)	2.5819 (18)	164.7 (18)
O4—H4⋯O3^i^	0.89 (3)	1.88 (3)	2.769 (3)	173 (3)

Symmetry code: (i) 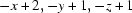.

**Fig. 1.**
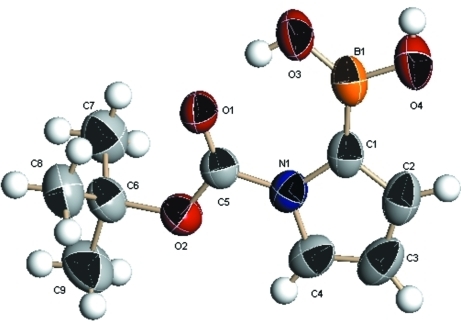
The molecular structure of the title compound with displacement ellipsoids drawn at the 50% probability level. H atoms are drawn as spheres of arbitrary radii.

## supplementary crystallographic information

### Comment

Boronic acids are versatile compounds widely used in the synthesis of biaryls, as therapeutical agents, and as chemical sensors (Hall, 2005). The title compound is the key intermediate for the synthesis of (+)-pinanediol-*L*-boroproline (Kelly & Fuchs, 1993).

In the molecular structure of the title compound (Fig. 1), the pyrrole ring, the boronic acid group and the carboxyl groups are almost co-planar. The carbonyl links with the adjacent boronic acid group *via* O3—H3···O2 hydrogen bonding. Intermolecular hydrogen bond is also observed in the crystal structure (Table 1).

### Experimental

All chemical reagents are commercial and used as received. Under -78°C and argon atmosphere, lithium diisopropylamide (1.0 *M* in THF, 5.0 ml, 5.0 mmol) was added dropwise to a solution of *tert*-butyl 1*H*-pyrrole-1-carboxylate (835 mg, 5.0 mmol) in dry THF (15 ml), and the solution was stirred at this temperature for 30 min. Trimethylborate (1.7 ml, 15 mmol) was added dropwise, and the mixture was allowed to warm to room temperature over 2 h and stirred overnight. After aqueous workup, the crude product was crystallized from hexanes. Single crystals suitable for X-ray analysis were obtained by recrystallization from a mixed solvent of ethyl acetate and hexane at ambient temperature (20–25°C).

### Refinement

H atoms were located in a difference Fourier map and refined isotropically.

### Crystal data

**Table:**

C_9_H_14_BNO_4_	*F*(000) = 896
*M**_r_* = 211.02	*D*_x_ = 1.244 Mg m^−^^3^
Orthorhombic, *P**b**c**a*	Mo *K*α radiation, λ = 0.71073 Å
Hall symbol: -P 2ac 2ab	Cell parameters from 810 reflections
*a* = 13.014 (3) Å	θ = 2.3–22.4°
*b* = 9.940 (2) Å	µ = 0.10 mm^−^^1^
*c* = 17.417 (4) Å	*T* = 293 K
*V* = 2252.9 (9) Å^3^	Prism, colorless
*Z* = 8	0.25 × 0.12 × 0.10 mm

### Data collection

**Table:**

Bruker SMART 1000 CCD area-detector diffractometer	1208 reflections with *I* > 2σ(*I*)
Radiation source: fine-focus sealed tube	*R*_int_ = 0.065
graphite	θ_max_ = 26.0°, θ_min_ = 2.3°
φ and ω scans	*h* = −16→13
9542 measured reflections	*k* = −12→12
2213 independent reflections	*l* = −21→19

### Refinement

**Table:**

Refinement on *F*^2^	Primary atom site location: structure-invariant direct methods
Least-squares matrix: full	Secondary atom site location: difference Fourier map
*R*[*F*^2^ > 2σ(*F*^2^)] = 0.042	Hydrogen site location: inferred from neighbouring sites
*wR*(*F*^2^) = 0.106	All H-atom parameters refined
*S* = 0.85	*w* = 1/[σ^2^(*F*_o_^2^) + (0.0588*P*)^2^] where *P* = (*F*_o_^2^ + 2*F*_c_^2^)/3
2213 reflections	(Δ/σ)_max_ < 0.001
192 parameters	Δρ_max_ = 0.12 e Å^−^^3^
0 restraints	Δρ_min_ = −0.20 e Å^−^^3^

### Special details

**Table:**

Geometry. All e.s.d.'s (except the e.s.d. in the dihedral angle between two l.s. planes) are estimated using the full covariance matrix. The cell e.s.d.'s are taken into account individually in the estimation of e.s.d.'s in distances, angles and torsion angles; correlations between e.s.d.'s in cell parameters are only used when they are defined by crystal symmetry. An approximate (isotropic) treatment of cell e.s.d.'s is used for estimating e.s.d.'s involving l.s. planes.
Refinement. Refinement of *F*^2^ against ALL reflections. The weighted *R*-factor *wR* and goodness of fit *S* are based on *F*^2^, conventional *R*-factors *R* are based on *F*, with *F* set to zero for negative *F*^2^. The threshold expression of *F*^2^ > σ(*F*^2^) is used only for calculating *R*-factors(gt) *etc*. and is not relevant to the choice of reflections for refinement. *R*-factors based on *F*^2^ are statistically about twice as large as those based on *F*, and *R*- factors based on ALL data will be even larger.

### Fractional atomic coordinates and isotropic or equivalent isotropic displacement parameters (Å^2^)

**Table:**

	*x*	*y*	*z*	*U*_iso_*/*U*_eq_	
B1	1.03949 (15)	0.6990 (2)	0.51802 (11)	0.0727 (6)	
C1	1.09811 (14)	1.0494 (2)	0.59196 (12)	0.0767 (5)	
C2	1.17519 (16)	1.0274 (3)	0.54329 (13)	0.0893 (7)	
C3	1.16298 (14)	0.8995 (3)	0.51194 (12)	0.0821 (6)	
C4	1.07732 (12)	0.83956 (18)	0.54116 (8)	0.0644 (5)	
C5	0.94986 (12)	0.92187 (19)	0.63777 (9)	0.0635 (4)	
C6	0.85439 (15)	1.03344 (19)	0.74024 (10)	0.0771 (5)	
C7	0.75166 (18)	1.0282 (3)	0.70204 (16)	0.0955 (7)	
C8	0.8711 (3)	0.9215 (3)	0.79700 (14)	0.1000 (7)	
C9	0.8746 (3)	1.1693 (3)	0.7756 (2)	0.1119 (9)	
N1	1.03646 (9)	0.93560 (14)	0.59221 (7)	0.0631 (4)	
O1	0.93714 (8)	1.02801 (13)	0.68119 (7)	0.0754 (4)	
O2	0.89519 (9)	0.82427 (13)	0.63724 (7)	0.0776 (4)	
O3	0.95566 (10)	0.63485 (15)	0.54641 (8)	0.0921 (5)	
O4	1.09809 (10)	0.6338 (2)	0.46635 (8)	0.0960 (5)	
H3	1.2044 (14)	0.8510 (17)	0.4741 (10)	0.083 (5)*	
H7A	0.7006 (17)	1.040 (2)	0.7397 (13)	0.104 (7)*	
H7B	0.7430 (18)	1.106 (3)	0.6664 (14)	0.131 (10)*	
H8A	0.8247 (16)	0.934 (2)	0.8403 (14)	0.109 (7)*	
H1	1.0760 (14)	1.126 (2)	0.6241 (11)	0.089 (6)*	
H9A	0.8259 (16)	1.184 (2)	0.8132 (14)	0.122 (8)*	
H9B	0.8706 (15)	1.236 (2)	0.7359 (14)	0.109 (9)*	
H7C	0.7384 (15)	0.942 (2)	0.6764 (13)	0.104 (7)*	
H2	1.2262 (17)	1.090 (2)	0.5323 (11)	0.104 (7)*	
H8B	0.941 (2)	0.928 (2)	0.8167 (14)	0.130 (9)*	
H9C	0.949 (2)	1.169 (3)	0.7937 (16)	0.169 (12)*	
H8C	0.8565 (17)	0.826 (3)	0.7735 (14)	0.135 (9)*	
H3A	0.9265 (14)	0.6888 (19)	0.5791 (11)	0.087 (6)*	
H4	1.080 (2)	0.548 (3)	0.4581 (16)	0.145 (12)*	

### Atomic displacement parameters (Å^2^)

**Table:**

	*U*^11^	*U*^22^	*U*^33^	*U*^12^	*U*^13^	*U*^23^
B1	0.0572 (11)	0.1046 (17)	0.0563 (11)	0.0146 (11)	−0.0040 (9)	−0.0053 (11)
C1	0.0616 (11)	0.0916 (15)	0.0767 (12)	−0.0077 (11)	−0.0134 (10)	0.0135 (12)
C2	0.0590 (12)	0.120 (2)	0.0888 (15)	−0.0117 (12)	−0.0074 (11)	0.0335 (14)
C3	0.0565 (11)	0.1260 (19)	0.0638 (11)	0.0091 (12)	0.0003 (9)	0.0161 (13)
C4	0.0524 (9)	0.0915 (13)	0.0492 (8)	0.0113 (9)	−0.0034 (7)	0.0069 (9)
C5	0.0586 (10)	0.0744 (12)	0.0576 (9)	0.0032 (9)	−0.0030 (8)	0.0004 (9)
C6	0.0834 (13)	0.0832 (13)	0.0647 (11)	0.0110 (10)	0.0065 (9)	−0.0149 (10)
C7	0.0793 (15)	0.116 (2)	0.0913 (17)	0.0166 (14)	0.0054 (13)	−0.0170 (17)
C8	0.119 (2)	0.115 (2)	0.0655 (13)	0.0172 (16)	0.0126 (14)	0.0019 (14)
C9	0.129 (2)	0.103 (2)	0.103 (2)	0.0076 (16)	0.0117 (18)	−0.0376 (18)
N1	0.0494 (7)	0.0814 (10)	0.0585 (8)	−0.0007 (7)	−0.0031 (6)	0.0075 (7)
O1	0.0787 (8)	0.0766 (8)	0.0708 (7)	−0.0010 (6)	0.0069 (6)	−0.0128 (7)
O2	0.0698 (7)	0.0786 (9)	0.0844 (8)	−0.0067 (6)	0.0214 (6)	−0.0166 (7)
O3	0.0730 (8)	0.1037 (11)	0.0996 (10)	−0.0004 (7)	0.0200 (7)	−0.0384 (8)
O4	0.0799 (9)	0.1217 (14)	0.0863 (9)	0.0147 (8)	0.0190 (7)	−0.0238 (9)

### Geometric parameters (Å, °)

**Table:**

B1—O4	1.346 (2)	C6—C7	1.494 (3)
B1—O3	1.357 (2)	C6—C8	1.504 (3)
B1—C4	1.535 (3)	C6—C9	1.507 (3)
C1—C2	1.331 (3)	C7—H7A	0.94 (2)
C1—N1	1.387 (2)	C7—H7B	0.99 (3)
C1—H1	0.99 (2)	C7—H7C	0.98 (2)
C2—C3	1.393 (3)	C8—H8A	0.97 (2)
C2—H2	0.93 (2)	C8—H8B	0.98 (3)
C3—C4	1.363 (3)	C8—H8C	1.05 (3)
C3—H3	0.978 (18)	C9—H9A	0.92 (2)
C4—N1	1.409 (2)	C9—H9B	0.96 (2)
C5—O2	1.2030 (19)	C9—H9C	1.02 (3)
C5—O1	1.309 (2)	O3—H3A	0.87 (2)
C5—N1	1.385 (2)	O4—H4	0.89 (3)
C6—O1	1.490 (2)		
			
O4—B1—O3	118.2 (2)	C6—C7—H7A	108.5 (13)
O4—B1—C4	115.60 (19)	C6—C7—H7B	110.6 (14)
O3—B1—C4	126.14 (17)	H7A—C7—H7B	105.0 (18)
C2—C1—N1	107.7 (2)	C6—C7—H7C	112.9 (12)
C2—C1—H1	135.0 (12)	H7A—C7—H7C	107.4 (18)
N1—C1—H1	117.2 (11)	H7B—C7—H7C	112 (2)
C1—C2—C3	108.3 (2)	C6—C8—H8A	109.2 (12)
C1—C2—H2	123.9 (13)	C6—C8—H8B	108.4 (15)
C3—C2—H2	127.8 (13)	H8A—C8—H8B	107 (2)
C4—C3—C2	110.3 (2)	C6—C8—H8C	112.7 (13)
C4—C3—H3	119.1 (11)	H8A—C8—H8C	107.5 (18)
C2—C3—H3	130.7 (10)	H8B—C8—H8C	111 (2)
C3—C4—N1	104.36 (18)	C6—C9—H9A	107.9 (16)
C3—C4—B1	124.22 (18)	C6—C9—H9B	108.3 (13)
N1—C4—B1	131.40 (15)	H9A—C9—H9B	112 (2)
O2—C5—O1	125.44 (15)	C6—C9—H9C	107.0 (17)
O2—C5—N1	123.79 (16)	H9A—C9—H9C	116 (2)
O1—C5—N1	110.77 (15)	H9B—C9—H9C	106 (2)
O1—C6—C7	109.75 (16)	C5—N1—C1	123.57 (16)
O1—C6—C8	108.80 (16)	C5—N1—C4	127.01 (15)
C7—C6—C8	113.4 (2)	C1—N1—C4	109.41 (16)
O1—C6—C9	100.86 (18)	C5—O1—C6	121.30 (13)
C7—C6—C9	111.6 (2)	B1—O3—H3A	107.4 (12)
C8—C6—C9	111.6 (2)	B1—O4—H4	114.5 (18)

### Hydrogen-bond geometry (Å, °)

**Table:**

*D*—H···*A*	*D*—H	H···*A*	*D*···*A*	*D*—H···*A*
O4—H4···O3^i^	0.89 (3)	1.88 (3)	2.769 (3)	173 (3)
O3—H3A···O2	0.87 (2)	1.73 (2)	2.5819 (18)	164.7 (18)

Symmetry codes: (i) −*x*+2, −*y*+1, −*z*+1.
